# Ultrasensitive electrochemical genosensors for species-specific diagnosis of malaria

**DOI:** 10.1016/j.electacta.2022.140988

**Published:** 2022-10-10

**Authors:** Felix Ansah, Francis Krampa, Jacob K. Donkor, Caleb Owusu-Appiah, Sarah Ashitei, Victor E. Kornu, Reinhard K. Danku, Jersley D. Chirawurah, Gordon A. Awandare, Yaw Aniweh, Prosper Kanyong

**Affiliations:** a West African Centre for Cell Biology of Infectious Pathogens (WACCBIP), College of Basic and Applied Sciences, University of Ghana, Legon, Accra, Ghana; b Department of Biochemistry, Cell and Molecular Biology, College of Basic and Applied Sciences, University of Ghana, Legon, Accra, Ghana; c Department of Chemical Engineering and Biotechnology, University of Cambridge, Philippa Fawcett Drive, Cambridge,CB3 0AS, United Kingdom; d Siemens Healthineers, Siemens Healthcare Diagnostics Products Ltd, Llanberis, Gwynedd LL55 4EL, United Kingdom

**Keywords:** Genosensor, Electrochemical impedance spectroscopy (EIS), Malaria, Plasmodium species, Deoxyribonucleic acid (DNA)

## Abstract

The absence of reliable species-specific diagnostic tools for malaria at point-of-care (POC) remains a major setback towards effective disease management. This is partly due to the limited sensitivity and specificity of the current malaria POC diagnostic kits especially in cases of low-density parasitaemia and mixed species infections. In this study, we describe the first label-free DNA-based genosensors based on electrochemical impedance spectroscopy (EIS) for species-specific detection of *P. falciparum, P. malariae* and *P. ovale*. The limits of detection (LOD) for the three species-specific genosensors were down in attomolar concentrations ranging from 18.7 aM to 43.6 aM, which is below the detection limits of previously reported malaria genosensors. More importantly, the diagnostic performance of the three genosensors were compared to quantitative real-time polymerase chain reaction (qPCR) assays using purified genomic DNA and the paired whole blood lysates from clinical samples. Remarkably, all the qPCR-positive purified genomic DNA samples were correctly identified by the genosensors indicating 100% sensitivity for each of the three malaria species. The specificities of the three genosensors ranged from 66.7% to 100.0% with a Therapeutic Turnaround Time (TTAT) within 30 min, which is comparable to the TTAT of current POC diagnostic tools for malaria. This work represents a significant step towards the development of accurate and rapid species-specific nucleic acid-based toolkits for the diagnosis of malaria at the POC.

## Highlights

• Electrochemical genosensors were developed for species-specific diagnosis of malaria.
• The genosensors are the first biosensors for detecting *P. malariae* and *P. ovale*.
• The limits of detection of the malaria genosensors are in the attomolar range.
• The genosensors can directly detect parasite nucleic acid without pre-amplification.
• The performance of the genosensors is comparable to real-time PCR.

## 1 Introduction

Human malaria remains a major global health concern despite the introduction of several interventions and control measures over the past two decades [1]. In 2020, the same number of malaria cases of 241 million were reported compared to malaria cases recorded in 2000 [1]. Five distinct *Plasmodium* species have been implicated in human malaria, namely, *Plasmodium falciparum, Plasmodium vivax, Plasmodium malariae, Plasmodium ovale* and *Plasmodium knowlesi* [2], howbeit, *Plasmodium cynomolgi* has recently been reported to infect humans [3,4]. In sub-Saharan Africa, the region that disproportionately bears high burden of global malaria cases (95%), *P. falciparum* accounts for majority of malaria morbidity and mortality, while the “minor” *P. malariae* and *P. ovale* are usually detected as low-density infections and often occur in co-infection with *P. falciparum* [1,5].

Over the past decades, malaria interventions and control measures have mainly focused on *P. falciparum* and *P. vivax* with the other non-falciparum species, largely neglected partly due to their marginal contribution to global malaria burden [[5], [6], [7]]. However, recent studies have highlighted the clinical significance of these non-falciparum species [[8], [9], [10]]. Both *P. malariae* and *P. ovale* have been implicated in major disease presentations such as severe anaemia, kidney-related complications, respiratory distress, hypotension, severe thrombocytopenia, jaundice, hepatomegaly, and hepatic dysfunction with possible fatal outcomes [9,[11], [12], [13], [14], [15]]. In addition, recent reports indicate an increasing prevalence of *P. malariae* and *P. ovale* in settings where *P. falciparum* transmission is decreasing [8,[16], [17], [18]].

Currently, the readily available POC diagnostic tools for malaria, including microscopy and rapid diagnostic tests (RDTs), lack adequate sensitivity and specificity for species-specific detection which is a major setback for the recommendation of appropriate antimalarial drugs and disease management [[19], [20], [21]]. This limitation calls for the availability of cost-effective, easy-to-use, and rapid diagnostic tools at the POC, especially, in resource-limited settings such as sub-Saharan Africa. In recent years, the application of biosensors, particularly the electrochemical-based ones, as diagnostic devices at the POC has received considerable attention [22]. Electrochemical-based biosensors have high sensitivity and specificity, quick turnaround time, low operational cost, and require less operational expertise [22,23].

Several biosensors have been reported for malaria diagnosis using various parasite biomarkers including antigens, antibodies, nucleic acids and infected red blood cells [24]. Amongst these biomarkers, parasite antigens are the most widely used, with *P. falciparum* histidine-rich protein 2 (PfHRP2) and genus *Plasmodium* lactate dehydrogenase (pLDH) being the most common analytes [25]. However, the reliability of these biomarkers has been limited by their global genetic diversity for both PfHRP2 and pLDH, and the persistence of PfHRP2 in the blood several days after antimalarial treatment [[26], [27], [28]]. In addition, these biomarkers lack specificity for species-specific detection of non-falciparum species.

Alternatively, nucleic acid-based biomarkers provide superior analytical performance for disease diagnosis [29]. Nucleic acid-based biorecognition receptors are relatively easy to identify, cost-effective and have high stability. Despite these advantages, only a few nucleic acid-based biosensors (genosensors) have been reported for the detection of *Plasmodium* species [24]. The first malaria genosensor was developed for the detection of *P. falciparum* based on quartz crystal microbalance (QCM) technology [30]. To reduce the operational cost, the same group described another QCM DNA-based genosensors for the detection of *P. falciparum* and *P. vivax* [31]. However, these genosensors require initial PCR amplification of the target DNA, which limits their application for routine diagnosis at the POC. As such, Ngo et al. developed a new genosensor based on surface-enhanced Raman scattering (SERS) for rapid detection of *P. falciparum* without the need for pre-amplification of target DNA [32]. Despite its high sensitivity, SERS-based biosensors require cumbersome multiple steps, large sample volume, and reagents which render them less suitable for routine POC applications [33].

In this study, we describe the first label-free DNA-based biosensors (genosensors) based on faradaic electrochemical impedance spectroscopy (EIS) for species-specific detection of *P. falciparum, P. malariae* and *P. ovale* using micro-gold electrode (µAuE). Amongst the various electrochemical detection technologies, label-free electrochemical impedance spectroscopy (EIS) represents a sensitive, convenient, and scalable tool for meeting the requirements of a cheap mass-produced diagnostic device. In brief, EIS technique involves the application of a small-amplitude sinusoidal potential wave on a DC potential biased electrode and monitoring the resulting current. By measuring the difference in amplitude and phase angle of potential and current sinusoids (the real and imaginary elements of the impedance) over a wide range of frequencies, the electrical properties of an electrode-electrolyte interface can be mapped to resolve time-dependant physical processes [34,35]. In Faradaic EIS, the properties of an interface are generally characterised by the solution resistance (R_s_), double-layer capacitance (_Cdl_) and charge-transfer resistance (R_ct_). These parameters can be modelled and quantified using an electrical equivalent circuit. Unlike SERS, EIS enables the development of scalable diagnostic devices using cheaper reagents, if any reagent is required, for ultrasensitive detection of target analytes [[34], [35], [36]]. This study demonstrates the potential application of EIS-based genosensors for rapid, accurate and cost-effective species-specific diagnosis of malaria at the POC.

## 2 Materials and methods

### 2.1 Materials and reagents

Sputtered chips consisting of micro-gold electrodes (µAuE) (*Ø* = ∼700 µm) were purchased from FlexMedical Solutions (Scotland, UK). Chemical reagents used in the study include 4-(2-hydroxyethyl)−1-piperazineethanesulfonic acid (HEPES), sodium bicarbonate (NaHCO_3_), phosphate-buffered saline (PBS) tablets, 6-mercapto-1-hexanol (MCH), and potassium ferrocyanide (K_4_[F*e*(CN)_6_]). All chemical reagents were of analytical grade and were purchased from Sigma Aldrich, UK. Hellmanex III cleaning solution was purchased from Sigma Aldrich, UK. Milli-Q ultra-pure water was obtained from the Millipore Milli-Q Integral Water Purification System (Millipore Corporation, Massachusetts, USA). The detection probes and the oligonucleotides were purchased from Sigma Aldrich, UK.

### 2.2 Characterisation of micro-gold electrode (µAuE)

**Scheme 1:**
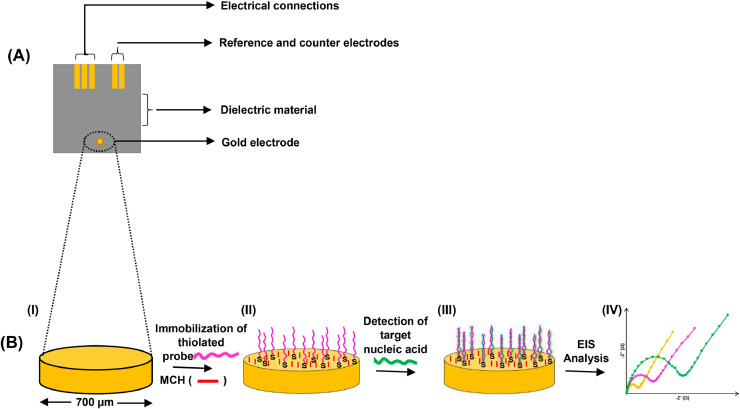
**The architecture of the Au chip and the schematic representation of the workflow.** **(A)** The architecture of the chip consisting of an electrical connection section, reference electrode, counter electrode and an insulating dielectric material (ash) that defines the working area of the µAuE. **(B) (I)** Extended view of the working electrode (µAuE) with a diameter of ∼700 µm. **(II)** The bare electrode was incubated with detection probe solution containing a mixture of the thiolated detection probe (magenta) and MCH blocking agent (red). **(III)** Incubation of the modified electrode with the target DNA (green). (**IV**) The resulting electrode was analysed using EIS. The expected Nyquist plots for the bare electrode (yellow), Au/probe/MCH electrode (magenta) and Au/probe/MCH/DNA_target_ electrode (green) following the stepwise modification. MCH = 6-mercapto-1-hexanol and EIS = electrochemical impedance spectroscopy.

Electrochemical experiments were carried out on the PGSTAT204 Autolab Potententat/Galvanostat/EIS FRAM32 Module (Metrohm-Atolab, Netherlands). The architecture of the sputtered chip consisting of the µAuE is illustrated in Scheme 1A. To characterise the bare electrodes, the chips were washed in 30% Hellmanex III solution for 30 min at room temperature, rinsed three times in excess Milli-Q water, and dried under a gentle stream of nitrogen gas. Following the cleaning of the electrodes, differential pulse voltammetry (DPV), cyclic voltammetry (CV) and EIS analysis were performed in 5.0 mM [Fe(CN)_6_]^3–^/[F*e*(CN)_6_]^4–^ prepared in 10 mM PBS (pH = 7.4). EIS response was carried out at 0.16 V over a frequency range of 100 kHz - 0.1 Hz.

### 2.3 Design of detection probes

The genomic sequence of the 18S rRNA gene of *P. falciparum* (XR_002273101.1), *P. malariae* (M54897.1), *P. ovale curtisi* (KF696371.1), *P. ovale wallikeri* (KF696364.1), and *P. vivax* (XR_003001225.1) were retrieved from the National Centre for Biotechnology Information (NCBI) database and aligned using Clustal Omega (https://www.ebi.ac.uk/Tools/msa/clustalo/, last accessed on 3rd March 2022). Conserved genomic regions specific to *P. falciparum, P. malariae* and *P. ovale* were selected to design the detection probes for each of the three *Plasmodium* species. The sequences of the detection probes have been shown in Table S1.

### 2.4 Immobilisation of detection probes

The fabrication of the genosensors was carried out following previously described experimental procedures [37,38] with few modifications. Briefly, following the successful characterisation of the bare electrodes, the detection probes were self-assembled on the electrodes using 10 µL of the probe solution containing 1.0 µM thiol-modified species-specific detection probe and 1.0 mM MCH solution (10 mM PBS, pH = 7.4) and then incubated overnight at 4℃ in a humidified chamber. Each of the species-specific modified electrodes was then rinsed in excess Milli-Q water to remove excess and/or unbound probes and MCH. EIS analysis was then performed for the modified electrodes (Au/probe/MCH) in 5.0 mM [Fe(CN)_6_]^3–^/[F*e*(CN)_6_]^4–^ as earlier described in this study. The *Plasmodium* species-specific modified electrodes were then rinsed in excess Milli-Q water and stored in sterile 10 mM PBS solution (pH = 7.4) at 4℃ until ready for use. The sequences of the thiol-modified DNA detection probes for *P. falciparum, P. malariae* and *P. ovale* are shown in Table S1.

### 2.5 Analytical performance of genosensors

Using optimal conditions, the limit of blank (LoB), limit of detection (LOD), the sensitivity and the specificity of the *P. falciparum, P. malariae* and *P. ovale* genosensors were determined. The LOD and sensitivity were determined using oligonucleotides that are complementary to the detection probe (cDNA) for each of the three *Plasmodium* species (Table S1). Briefly, the species-specific cDNA oligonucleotides were serially diluted in nuclease-free water to concentrations in the range of 10 aM - 320 aM. A volume of 10 µL of each diluted cDNA concentration was incubated at 95 ℃ for 5 min and then applied to their corresponding modified electrodes (Au/probe/MCH) at 37 ℃ for 15 min. Following the incubation, the electrodes were washed in excess Milli-Q water and dried under a gentle stream of nitrogen gas, and EIS spectra acquired. The resulting spectra were analysed using Bode plots. The normalised R_ct_ values (Relative Response, RR) of the genosensors were calculated using the equation RR = [(R_1_ - R_0_)/ R_0_], where R_0_ and R_1_ represent the charge transfer resistance (R_ct_) before and after addition of the cDNA, respectively, obtained within the frequency range of 100 kHz - 0.1 Hz. The RR values were plotted against the log_10_-transformed concentrations of the cDNA to obtain a calibration plot from which the LOD and sensitivity were estimated. The LOD and sensitivity were determined using the formula 3.3x σ/*S* and *S*/A, respectively, where σ is the standard deviation of the blanks, *S* is the slope of the calibration curve, and *A* is the surface area of the µAuE. The LoB for each of the genosensors was determined using 10 mM PBS (pH = 7.4). The limit of blank RR (RR_LoB_) of the three genosensors were obtained using the formula, RR_LoB_ = x̄_Blank_ + 3σ, where x̄_Blank_ is the mean RR of the blanks and σ is the standard deviation of the blanks.

To determine the specificity, the species-specific modified electrodes (Au/probe/MCH) were independently incubated with 10 µL of 100 aM of the target cDNA, 3 base-pair mismatch and non-complementary oligonucleotides at 37 ℃ for 15 min. The resulting electrodes were washed in excess Milli-Q water and dried under a gentle stream of nitrogen gas. EIS spectra were then acquired to determine the relative response (RR). The sequences of the cDNA, the 3 base-pair mismatch and the non-complementary oligonucleotides are shown in Table S1.

### 2.6 Species-specific detection of P. falciparum, P. malariae and P. ovale in clinical isolates

To validate the genosensors for the detection of *Plasmodium* parasites in clinical isolates, a total of 24 samples were selected from a large pool of cryopreserved clinical isolates that have been analysed using quantitative real-time polymerase chain reaction (qPCR) in our previous study [8]. The 24 samples that were selected based on the qPCR results consisted of twelve samples for *P. falciparum* assay (nine positives and three negatives), six samples for *P. malariae* assay (three positives and three negatives), and six samples for *P. ovale* assay (three positives and three negatives). Each of the 24 samples had purified genomic DNA and the paired whole blood samples.

#### 2.6.1 qPCR detection of plasmodium species

The details of the SYBR Green-based qPCR assays have been described in our initial study [8]. Briefly, all reactions were performed on the QuantStudio5 system (Applied Biosystems, UK) in a total volume of 15 µL containing 1X Luna Universal qPCR Master Mix (New England BioLabs, UK), 250 nM of each of the forward and the reverse primers and 3 µL of the template DNA. The cycling conditions for the assays consisted of 3 min at 95℃ followed by 45 cycles of 15 s at 95℃, 40 s at 50℃ and 40 s at 60℃. The specificity of the qPCR products was determined using the resulting melting curves.

#### 2.6.2 Detection of plasmodium species using genosensors

For purified DNA samples, genomic DNA was purified from 200 µL of the whole blood using the QIAamp DNA Extraction Mini Kit (Qiagen, UK) and eluted with 100 µL elution buffer following the instructions from the manufacturer. The purified genomic DNA samples were incubated at 95 ℃ for 10 min and immediately placed on ice. The heating process enables denaturation of the double-stranded genomic DNA, while the ice limits immediate renaturation of the denatured genomic DNA. A volume of 10 µL of each of the denatured DNA samples was applied to the species-specific modified electrodes (Au/probe/MCH) and incubated at 37 ℃ for 15 min. The electrodes were then washed in excess Milli-Q water and then dried under a gentle stream of nitrogen gas. Using the EIS analysis, the relative response (RR) of the genosensors were determined as described earlier in this study.

Lastly, the direct use of clinical samples without DNA pre-purification was investigated. To achieve this, a volume of 10 µL whole blood was diluted with 90 µL of lysis buffer (Qiagen, UK). The resulting whole blood lysate was incubated at 95 ℃ for 10 min and immediately placed on ice. A volume of 10 µL of the lysate was then applied to the modified electrodes (Au/probe/MCH) and incubated at 37 ℃ for 15 min. The electrodes were washed in excess Milli-Q water, dried under a gentle stream of nitrogen gas and then EIS spectra acquired to determine the relative response (RR).

### 2.7 Ethical consideration

The study obtained an ethical approval from the ethics committees of the Ghana Health Service (GHSERC005/12/17), the Noguchi Memorial Institute for Medical Research, University of Ghana (NMIMR-IRB CPN 077/17–18) and the Kintampo Health Research Centre (KHRCIEC/2018–10). All participants and/or parents or guardians of participants gave a written informed consent prior to recruitment.

## 3 Results and discussion

To date, accurate detection of low-density *Plasmodium* parasite infections, especially *P. malariae* and *P. ovale* infections, remains a challenge as the most readily available POC malaria diagnostic tools which include microscopy and rapid diagnostic tests [27,[39], [40], [41]] have limited sensitivity and lack adequate specificity [20,42]. As such, nucleic acid-based amplification assays such as polymerase chain reaction (PCR) and loop-mediated isothermal amplification (LAMP) with high diagnostic performance have been described as more reliable tools for the detection of *Plasmodium* species [42,43]. However, these nucleic acid-based amplification tools require expensive reagents and high technical expertise, which render them less suitable to routine POC diagnosis in resource-limited settings [44]. In recent years, the application of biosensors as POC devices for malaria diagnosis is increasingly gaining interest [24,45]. In this study, we developed DNA-based label-free electrochemical genosensors for species-specific detection of *P. falciparum, P. malariae* and *P. ovale* in clinical isolates without the need for pre-amplification of the target DNA. Notably, the genosensors were used to directly detect parasite genomic DNA in clinical isolate lysates without purification of target nucleic acid material. This characteristic performance of the current genosensors offers several advantages, including the elimination of well-known challenges and risks associated with nucleic acid purification and pre-amplification steps.

### 3.1 The principle of detection of plasmodium genomic DNA

As shown in Scheme 1A, each chip consists of an electrical connector, an insulating dielectric material, reference electrode, counter electrode, and a working micro-gold electrode (µAuE). A stepwise schematic representation of the assay development workflow is shown in Scheme 1B Firstly, the impedance spectra of the cleaned bare micro-gold (Au) electrodes were determined. Following this, the bare electrodes were incubated with the detection probe solution containing the thiolated probe (1.0 µM) and the MCH solution (1.0 mM). Co-immobilisation approach of the probe and MCH was used since this process produces a consistent self-assembled monolayer on an electrode [37,38]. The MCH was used to block any exposed surface on the electrode to minimise non-specific interactions. Following the modification, the impedance spectra of the modified electrodes (Au/probe/MCH) were determined. The modified electrodes were then incubated with the target DNA or sample of interest. The target DNA, if present, interacts with the immobilised probe, which is characterised by an increase in R_ct_ since the flow of electrons produced by the [Fe(CN)_6_]^3–^/[F*e*(CN)_6_]^4–^ redox couple is hindered at the surface of the electrode [34,35]. As a result, higher R_ct_ value is expected in the presence of the target DNA (Au/probe/MCH/DNA_target_) compared to the R_ct_ value before the addition of the sample of interest (Au/probe/MCH). The resulting R_ct_ values are directly proportional to the concentrations of the captured target DNA. On the other hand, in the absence of the target DNA, no significant change in R_ct_ is expected before and after the addition of the sample of interest.

### 3.2 Characterisation of the micro-gold electrode

DPV, CV and EIS were used to determine the electrochemical properties of the bare electrode. From the DPV analysis, the peak current and the corresponding potential for the [Fe(CN)_6_]^3–^/[F*e*(CN)_6_]^4–^ redox couple were 2.27 ± 0.27 µA and 0.16 ± 0.01 V, respectively (Fig. S1A). The resulting average potential (0.16 V) was then used to acquire all the impedance spectra. Based on the CV analysis, an increase in current was observed with an increasing scan rate in the range of 10 mV/s - 300 mV/s (Fig. S1B). Good linear relationships were obtained for both the peak anodic and peak cathodic currents with coefficient of determination, R^2^ > 0.99 (Fig. S1C). The R_ct_ values recorded on different bare µAuE indicated that the electrodes were highly reproducible with an average correlation coefficient of 3.4% (Fig. S1D).

### 3.3 Analytical performance of genosensors

**Fig. 1:**
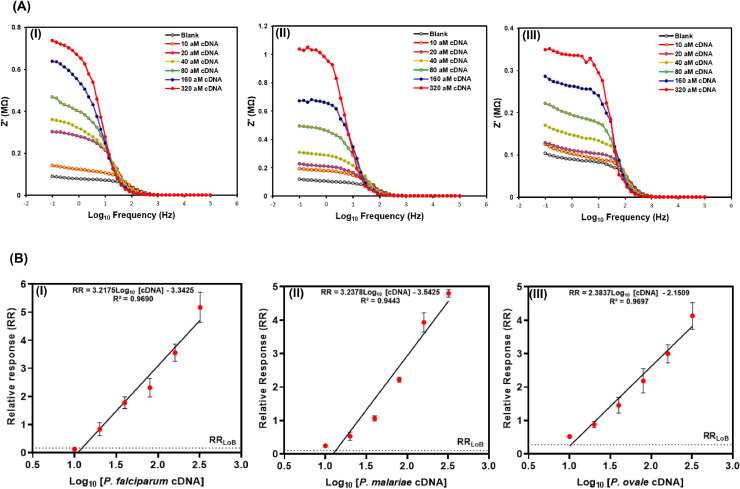
**The sensitivity of the genosensors**. **(A)** Bode plots for *P. falciparum***(I)**, *P. malariae***(II)** and *P. ovale***(III)** genosensors with cDNA concentrations ranging from 10.0 aM to 320.0 aM. Increasing impedance (Z’) were observed with increasing cDNA concentration for all three *Plasmodium* species. **(B)** The linear relationship between the relative response (RR) and the log_10_-transformed cDNA concentrations for *P. falciparum***(I)**, *P. malariae***(II)** and *P. ovale***(III)** obtained from their corresponding Bode plots (*n* = 3). The impedance spectra for determining the RR were acquired within the frequency range of 100 kHz - 0.1 Hz. Short-dashed lines represent limit of blanks (RR_LoB_).

The dose responses for the detection of *P. falciparum, P. malariae* and *P. ovale* are expressed in the Bode plots in Fig. 1A with their corresponding calibration plots in Fig. 1B. The sensitivity and the LOD of each species-specific genosensor were determined using cDNA concentrations in the range of 10 aM - 320 aM. Increasing impedance spectra were observed with increasing concentration of the cDNA for each of the three species-specific genosensors as shown in the Bode plots in Fig. 1A. There were good linear relationships between the relative response (RR) and the log_10_–transformed concentrations of the cDNA for all the three species-specific genosensors (Fig. 1B). The fitted linear regression equations for the *P. falciparum, P. malariae* and *P. ovale* genosensors were RR = 3.2175*Log_10_ [cDNA] - 3.3425 (R² = 0.9690), RR = 3.2378*Log_10_ [cDNA] - 3.5425 (R² = 0.9443), and RR = 2.3837*Log_10_ [cDNA] - 2.1509 (R² = 0.9697), respectively. Based on these linear relationships, the sensitivities for the genosensors were estimated to be 868.4 MΩ.aM^–1^.cm^–2^, 920.4 MΩ.aM^–1^.cm^–2^ and 558.8 MΩ. aM^–1^.cm^–2^ for *P. falciparum, P. malariae* and *P. ovale*, respectively (Table 1). The estimated LODs for the *P. falciparum, P. malariae* and *P. ovale* genosensors were 18.7 aM, 43.6 aM and 27.9 aM, respectively (Table 1). The RR for the limit of the blank (RR_LoB_) (represented as short-dashed lines in Fig. 1B) obtained using sterile PBS were RR_LoB_ = 0.17, RR_LoB_ = 0.10, and RR_LoB_ = 0.28 for *P. falciparum, P. malariae* and *P. ovale* genosensors, respectively. The LODs of these genosensors are comparable to previously reported DNA-based sensors, which were also in the attomolar range [[46], [47], [48]]. To the best of our knowledge, this study describes the first species-specific genosensors for malaria diagnosis. The observed LODs of the current genosensors are exceptionally low for unamplified label-free assays. These characteristics are notably superior, being the first impedance-based sensors for species-specific malaria diagnosis in clinical samples. This electrochemical platform, unlike those highlighted in Table 1, is a single-step and require no pre-amplification step; which could be readily integrated into both automated fluid handlers and multiplex formats with retention of assay performance characteristics. In addition, the use of synthetic nucleic acid detection probes would make the genosensors relatively less expensive for population-wide surveillance and routine POC diagnosis of malaria. Cost is a relevant point to be considered for the commercialisation of these devices especially in resource-limited malaria endemic settings such as sub-Saharan Africa.

**Table 1:** Comparison of the LODs of genosensors for the detection of *Plasmodium* species.

***Plasmodium* species**	**Nano-material**	**Detection technique**	**Initial pre-amplification**	**LOD**	**Sensitivity (MΩ.aM^–1^.cm^–2^)**	**References**
						
*P. falciparum*	Gold	QCM*	PCR	0.025 ng/mL	–	[30]
						
*P. falciparum*	Silver	QCM*	PCR	–	–	[31]
						
*P. vivax*	Silver	QCM*	PCR	–	–	[31]
						
*P. falciparum*	Magnetic beads and nano-rattles	SERS^#^	Not required	100 aM	–	[32]
						
*P. falciparum*	Gold	EIS	Not required	18.7 aM	868.4	Current study
						
*P. malariae*	Gold	EIS	Not required	43.6 aM	920.4	Current study
						
*P. ovale*	Gold	EIS	Not required	27.9 aM	558.8	Current study

LOD = Limit of detection, QCM* = Quartz crystal microbalance; SERS^#^ = Surface-enhanced Raman scattering, EIS = Electrochemical impedance spectroscopy, and “– ” represents unreported parameter.

**Fig. 2:**
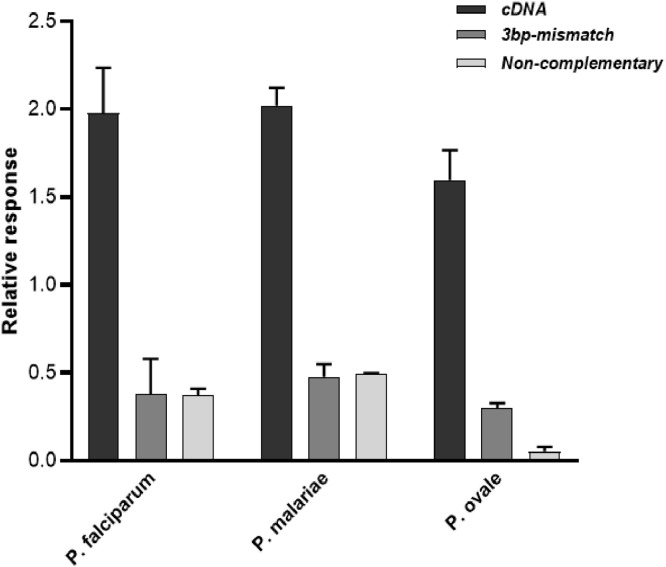
**The specificity of the genosensors.** The specificity of the *P. falciparum, P. malariae*, and *P. ovale* genosensors using 100 aM of complementary (cDNA), 3 base-pair (bp) mismatch and non-complementary oligonucleotides (*n* = 3).

For specificity, the relative response (RR) for the cDNA oligonucleotides for all the three species-specific genosensors were at least 4-fold higher than that of the non-complementary and the 3-base pair mismatch oligonucleotides (Fig. 2). The recovery rate of target cDNA was also assessed by spiking *Plasmodium*-negative whole blood sample with the target cDNA. The cDNA recovery rates (%) for the spiked lysates were 85.4%, 87.8% and 78.1% for *P. falciparum, P. malariae* and *P. ovale*, respectively (Table 2). The relative standard deviation (RSD) recorded for the three genosensors ranged from 2.06% to 8.16% (Table 2). A decrease in the relative response was observed for all the three species-specific genosensors when incubated with the unspiked *Plasmodium*-negative whole blood lysate (Table 2). This observation could be due to the electrical properties of blood and its constituents including haemoglobin and electrolytes (e.g. sodium and potassium ions) which could interact with the negatively charged phosphodiester backbone of the immobilised DNA probe [48,49].

**Table 2:** cDNA recovery from whole blood lysates spiked with 100 aM of target cDNA.

**Biosensor**	*Relative R**esponse (RR)**					**Recovery rate**	#**Recovery**	**RSD**
	cDNA only		cDNA-spiked lysate		Unspiked lysate	**(%)**	(aM)	**(%)**
*P. falciparum*	1.98 ± 0.26		1.69 ± 0.16		- 0.41 ± 0.02	85.4	85.4	8.16
*P. malariae*	2.05 ± 0.12		1.80 ± 0.08		- 0.94 ± 0.06	87.8	87.8	7.65
*P. ovale*	1.60 ± 0.17		1.25 ± 0.10		- 0.49 ± 0.06	78.1	78.1	2.06

⁎ The normalised relative response (RR) values are presented as average from replicates plus or minus standard deviation (*n* = 3).

# Recovery represents concentration of cDNA recovered from the lysates spiked with 100aM of target cDNA. RSD = relative standard deviation.

### 3.4 Detection of plasmodium species genomic DNA in clinical isolates

**Fig. 3:**
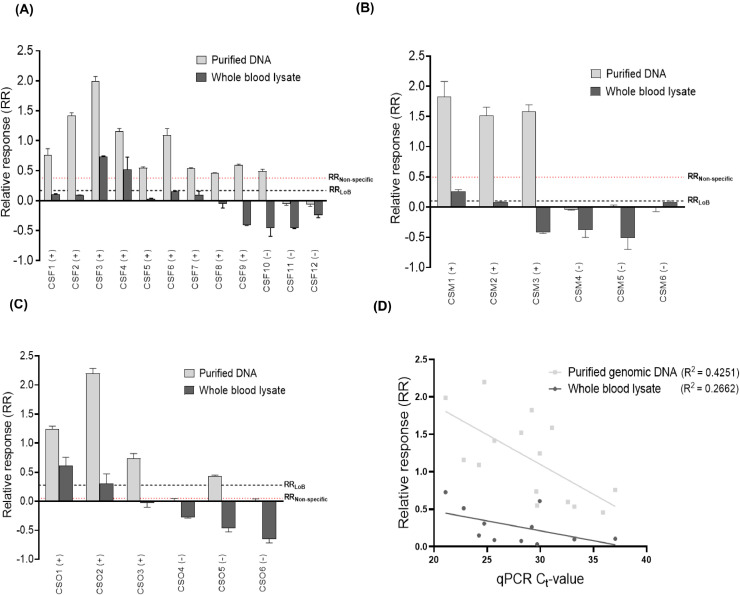
**Detection of Plasmodium species in purified genomic DNA and whole blood lysates obtained from clinical isolates.** **(A)** Relative response for *P. falciparum* genosensor for twelve clinical samples (CSF1 - CSF12). **(B)** Relative response for *P. malariae* genosensor for six clinical samples (CSM1 – CSM6). **(C)** Relative response for *P. ovale* genosensor for six clinical samples (CSO1 – CSO6). The symbols “+” and “-” represent qPCR results for positive and negative samples, respectively. Short-dashed lines labelled RR_LoB_ (Black) and RR_Non-specific_ (Red) represent the RR for the limit of the blank and non-specific oligonucleotide, respectively. The short-dashed lines were used as threshold values for determining positivity **(D)** Correlation analysis between qPCR C_t_-values and biosensor relative response. R^2^ represents the coefficient of determination, (*n* = 3).

The practicability of the genosensors for the detection of *P. falciparum, P. malariae* and *P. ovale* in clinical isolates was assessed using purified genomic DNA samples and the paired whole blood lysates that were selected from a previously qPCR-analysed sample pool [8]. Positivity was defined by an increase in the relative response (RR) following incubation of the modified electrode with the sample of interest. To identify positive samples, the limit of blank RR (RR_LoB_) was used as the baseline or threshold values for each of the three *Plasmodium* species. Clinical samples with RR above the threshold values were considered positive. Compared to the qPCR assays, the diagnostic sensitivity of the *P. falciparum, P. malariae* and *P. ovale* genosensors using purified genomic DNA samples were 100% (9/9), 100% (3/3) and 100% (3/3), respectively (Fig. 3A - 3C; Table S2). The diagnostic specificity of the *P. falciparum, P. malariae* and *P. ovale* genosensors for the purified genomic DNA samples were 66.7% (2/3), 100% (3/3) and 66.7% (2/3)*,* respectively (Fig. 3A - 3C; Table S2). Using the paired whole blood lysates, the diagnostic sensitivity of the *P. falciparum, P. malariae* and *P. ovale* genosensors were 22.2% (2/9), 33.3% (1/3) and 66.7% (2/3), respectively (Fig. 3A - 3C; Table S2). Also, the specificity for the whole blood lysates were 100% (3/3) for the three *Plasmodium* species-specific genosensors (Fig. 3A - 3C; Table S2); these are significantly higher than that achievable with microscopy [50], [51], [52], [53], [54] and rapid diagnostic tests (RDTs) for detection of low-density *Plasmodium* infections [40].

In redefining the threshold line using the RR of the non-specific (non-complementary) oligonucleotide (RR_Non-specific_) for *P. falciparum* (0.37), *P. malariae* (0.50) and *P. ovale* (0.05) as shown in Fig. 2, the purified DNA samples had the same diagnostic sensitivity (100%) and specificity (66.7 – 100%) (Fig. 3A - 3C). Similarly, the diagnostic performance obtained for the *P. falciparum* and *P. ovale* genosensors on the whole blood lysates using the RR of the non-complementary oligonucleotide as the threshold line were comparable to the diagnostic performance obtained with the RR_LoB_ as the threshold (Fig. 3A and 3C). The *P. malariae* genosensor, however, had reduced sensitivity of 0% (0/3) as the RR of the qPCR-positive samples were below the RR of the non-complementary oligonucleotide (Fig. 3B). It is relevant to highlight that the limited sample size used in this study may not reflect the actual diagnostic performance of the genosensors. As such, further studies involving a larger clinical sample size would be necessary to properly estimate the diagnostic performance of the genosensors relative to other established methods such as PCR.

We finally assessed parasite quantification using the genosensors by correlating the relative response (RR) with the qPCR threshold cycle (C_t_) values (Fig. 3D). The RR values moderately correlated with the qPCR C_t_-values with coefficient of determination, R^2^ = 0.425 for purified genomic DNA and R^2^ = 0.266 for whole blood lysates, indicating that the genosensors have the potential to quantitatively detect *Plasmodium* species in both purified DNA samples and whole blood lysates.

## 4 Conclusion

The current study developed DNA-based impedance sensors and demonstrated the practicability of the genosensors for species-specific detection of *P. falciparum, P. malariae* and *P. ovale* genomic DNA. When combined with the intrinsic scalability of EIS platform, the sensor design supports diagnosis of malaria down to exceptionally low levels with LODs in the attomolar range. More significantly, the study showed the feasibility of the genosensors for the detection of *Plasmodium* species in clinical isolates without the need for pre-purification of the target DNA. In addition, the results could be obtained within 30 min which is comparable to the therapeutic turnaround time (TTAT) for microscopy and RDTs, but lower than the TTAT for nucleic acid-based amplification assays such as PCR and LAMP. This study represents a significant step towards the development of species-specific POC sensors for malaria diagnosis.

## CRediT authorship contribution statement

**Felix Ansah:** Conceptualization, Methodology, Investigation, Data curation, Writing – original draft. **Francis Krampa:** Methodology. **Jacob K. Donkor:** Methodology. **Caleb Owusu-Appiah:** Methodology. **Sarah Ashitei:** Methodology. **Victor E. Kornu:** Methodology. **Reinhard K. Danku:** Methodology. **Jersley D. Chirawurah:** Methodology, Writing – review & editing. **Gordon A. Awandare:** Conceptualization, Writing – review & editing, Supervision, Funding acquisition. **Yaw Aniweh:** Conceptualization, Data curation, Writing – review & editing, Supervision. **Prosper Kanyong:** Conceptualization, Data curation, Writing – review & editing, Supervision.

## Declaration of Competing Interest

Gordon A. Awandare reports financial support was provided by National Institute for Health Research.

## Data Availability

Data will be made available on request.

## Appendix Supplementary materials

Image, application 1
