# Supplementary material for: Ultrasensitive electrochemical genosensors for species-specific diagnosis of malaria
Source: Electrochim Acta. 2022 Oct 10;429:140988. doi: 10.1016/j.electacta.2022.140988 (PMC9472471; doi:10.1016/j.electacta.2022.140988)
Supplement: Supplementary file 1 [file mmc1.docx]

**Supporting Information**

Felix Ansah^a, b^, Francis Krampa^c^, Jacob K. Donkor^b^, Caleb Owusu-Appiah^a^, Sarah Ashitei^a^, Victor E. Kornu^b^, Reinhard K. Danku^a^, Jersley D. Chirawurah^a, b^, Gordon A. Awandare^a, b^, Yaw Aniweh^a^, Prosper Kanyong^a, d, *^

^a^West African Centre for Cell Biology of Infectious Pathogens (WACCBIP), College of Basic and Applied Sciences, University of Ghana, Legon, Accra, Ghana

^b^Department of Biochemistry, Cell and Molecular Biology, College of Basic and Applied Sciences, University of Ghana, Legon, Accra, Ghana

^c^Department of Chemical Engineering and Biotechnology, University of Cambridge, Philippa Fawcett Drive, Cambridge, CB3 0AS, UK

^d^Siemens Healthineers, Siemens Healthcare Diagnostics Products Ltd, Llanberis, Gwynedd LL55 4EL, UK

***Corresponding Author:** Siemens Healthineers, Siemens Healthcare Diagnostics Products Ltd, Llanberis, Gwynedd LL55 4EL, UK. *E-mail address*: [p.kanyong@waccbip.org](mailto:p.kanyong@waccbip.org) (P. Kanyong).

**Table S1: List of detection probes and oligonucleotides for genosensor development.**

| **Target** | **DNA probe/Target sequence** | **Sequence** |
| --- | --- | --- |
| *P. falciparum* | Detection probe | 5'-HS-(CH_2_)_6_-GTAACTATTCTAGGGGAACTATTTTAGC-3' |
|  | Complementary | 5'-GCTAAAATAGTTCCCCTAGAATAGTTAC-3' |
|  | Three-base mismatch* | 5'-GCT**G**AAATAGTTC**A**CCTAGAATAGT**G**AC-3' |
|  |  |  |
| *P. malariae* | Detection probe | 5'-HS-(CH_2_)_6_-GTTGTACGTTAAGAATAACCGCCAAGGC-3' |
|  | Complementary | 5'-GCCTTGGCGGTTATTCTTAACGTACAAC-3' |
|  | Three-base mismatch* | 5'-GCC**C**TGGCGGTTAT**C**CTTAACGTAC**T**AC-3' |
|  |  |  |
| *P. ovale* | Detection probe | 5'-HS-(CH_2_)_6_-GATGCTTAGACAATACAACGTATCTG-3' |
|  | Complementary | 5'-CAGATACGTTGTATTGTCTAAGCATC-3' |
|  | Three-base mismatch* | 5'-CAG**C**TACGTTG**A**ATTGTCTAAGC**T**TC-3' |
|  | Non-complementary | 5’-AACCCAAAGACTTTGATTTCTCATAA-3’ |

*****The highlighted nucleotides (bold red) indicate mismatched bases that were introduced


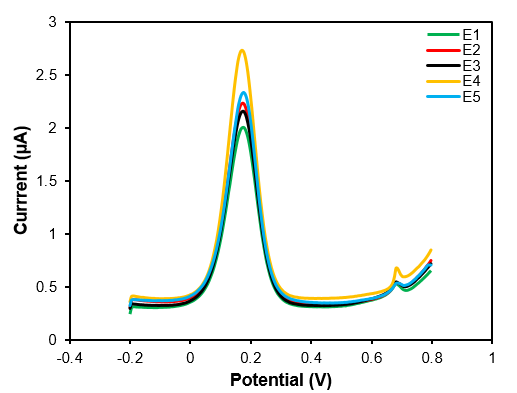

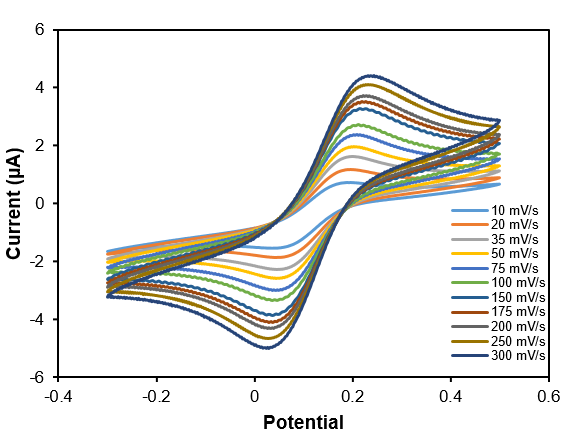

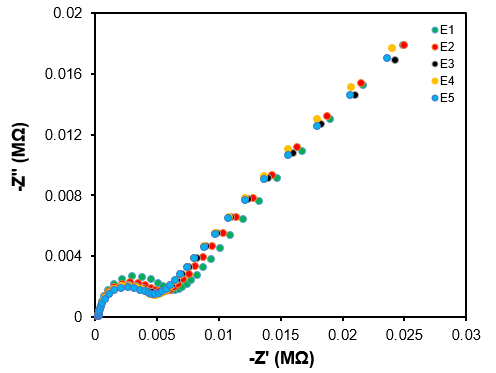

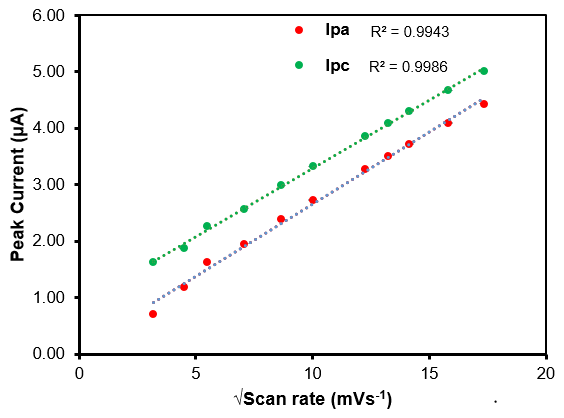


**(A)**

**(C)**

**(B)**

**(D)**

**Figure S1: Characterisation of the bare micro-gold electrode (µAuE).**

(**A**) DPVs for five different electrodes. (**B)** CVs for the bare electrode in 5.0 mM at different scan rates ranging from 10 mV/s to 300 mV/s. **(C)** Relationship between the peak anodic, Ipa (Red) and peak cathodic, Ipc (Green) currents as a function of the scan rate. A linear correlation was observed for both the peak anodic (Red) and peak cathodic (Indigo) currents with R^2^ > 0.99. **(D)** Nyquist plots showing the charge transfer resistance (R_ct_) of five independent electrodes. All measurements were recorded in 5.0 mM [Fe(CN)_6_]^-3/-4^ prepared in PBS (10 mM; pH 7.4).

**Table S2: The analytical performance of the biosensors compared to qPCR assays.**

| **Biosensor** | **Sample size** | **Sample type** | **Sensitivity (%)** | **Specificity (%)** |
| --- | --- | --- | --- | --- |
| *P. falciparum* | 12 | Purified genomic DNA  Whole blood lysate | 100  22.2 | 66.7  100 |
|  |  |  |  |  |
| *P. malariae* | 6 | Purified genomic DNA  Whole blood lysate | 100  33.3 | 100  100 |
|  |  |  |  |  |
| *P. ovale* | 6 | Purified genomic DNA  Whole blood lysate | 100  66.7 | 66.7  100 |
|  |  |  |  |  |
